# What is the impact of sports‐related gambling advertising on gambling behaviour? A systematic review

**DOI:** 10.1111/add.16761

**Published:** 2025-01-11

**Authors:** Ellen McGrane, Robert Pryce, Matt Field, Shangshang Gu, Esther C. Moore, Elizabeth Goyder

**Affiliations:** ^1^ Sheffield Centre for Health and Related Research (SCHARR) University of Sheffield Sheffield UK; ^2^ Department of Psychology University of Sheffield Sheffield UK

**Keywords:** advertising, behaviour, gambling, policy, public health, sports, systematic review

## Abstract

**Background and Aims:**

Gambling is a public health issue and widespread advertising of gambling products may contribute to gambling harms. Sports‐related gambling advertising includes advertising around sports games or for sports betting products. This review aimed to provide the most systematic and up‐to‐date review of the literature on the association between sports‐related gambling advertising and gambling behaviour.

**Methods:**

A systematic literature search of quantitative studies up to 13 February 2024 was undertaken following PRISMA guidelines. Inclusion criteria were quantitative studies published in the English language exploring the association between sports‐related gambling advertising and gambling behaviour. Traditional database searches (Medline, Scopus, PsychInfo, Web of Science, CINAHL and The Cochrane Library) were undertaken alongside citation, author and website searches. Studies were narratively synthesised, and the overall quality of the evidence was assessed using the Mixed Methods Appraisal Tool (MMAT).

**Results:**

Twenty‐two studies were included in this review covering traditional, digital, direct, embedded, inducement and aggregate advertising. The majority (*n* = 16) of research was undertaken in Australia on adult populations. Results suggest that sports‐related gambling advertising is associated with increases in perceived, intended and actual frequency of (*n* = 6 studies) and expenditure on (*n* = 3) gambling, unplanned or unintended gambling (*n* = 2), the likelihood of gambling (*n* = 2), the likelihood of using a sponsor's product (*n* = 2) and, in some cases, the complexity or riskiness of bets placed (*n* = 2). Studies suggest that the self‐reported effect may be more pronounced in higher‐risk gamblers (*n* = 7). Preliminary evidence suggests that specific inducements which reduce the riskiness or cost of gambling appear to be particularly influential (*n* = 3). Limitations of the evidence base include the lack of standardised measures and use of observational designs.

**Conclusions:**

Exposure to sports‐related gambling advertising appears to be associated with increased gambling behaviour for a wide range of advertising media. This association may be more pronounced in higher‐risk gamblers who are already at increased risk of harm.

## INTRODUCTION

Gambling advertising has attracted the interest of researchers and policymakers world‐wide, with studies citing its widespread and highly targeted nature [1, 2]. Rapid technological change over the last 20 years has increased accessibility to gambling [3]. Advertising has mirrored this trend, increasing in frequency and complexity [1, 2]. The potential increase in harms has garnered the attention of public health stakeholders. Harms from gambling can occur to the individual, affected others (e.g. family or friends) and to wider society [4, 5]. These encompass harms to resources (e.g. employment or debt), relationships (e.g. family or partner), health (physical or mental) [4] and other aspects (e.g. criminal or cultural) [5]. Harms may continue after individual gambling ceases; referred to as ‘legacy harms’ [5]. Consequently, gambling has been identified as a public health problem [6, 7, 8, 9, 10]. The impact of gambling advertising on gambling behaviour and subsequent harms has been the focus of research in recent years. The association between sports and gambling has received significant attention, with studies citing widespread prevalence on television, pitch‐side hoardings and via sponsorship deals [1, 2, 11, 12, 13, 14].

There are existing reviews on gambling advertising and its direct relationship with behaviour. A review [15] concluded that gambling advertising positively increases gambling attitudes, intentions and behaviours; the latter being statistically significant in a meta‐analysis. There is some evidence of a dose–response effect, whereby increased exposure to advertising is associated with an increasing effect on gambling behaviour. An umbrella review [16] and a review in the sporting context [17] identified similar results.

Despite the strong contributions of existing reviews, searches only go up to 2021 [15, 16, 17]. Two of these reviews take a broad approach, exploring the impact of all types of gambling advertising on behaviour [15, 16]. However, sports‐related advertising has received particular attention because of its widespread nature, potential to normalise gambling and create a gateway to gambling harm [18, 19, 20]. An existing, more focussed, review on this topic does not include studies on children or grey (unpublished) literature [17]. Including grey literature in a systematic review can help to minimise publication bias and foster a more balanced view of the evidence base [21, 22]. Given that public health research on gambling is less well‐developed compared to alcohol and tobacco, examining the grey literature may be important for obtaining a more thorough understanding of the evidence base.

There are other systematic reviews that focus on adjacent topics, such as the type of marketing strategies used by the gambling industry [23]. However, there has been no updated comprehensive systematic review exploring the direct association between sports‐related gambling advertising and gambling behaviour. In this review, sports‐related gambling advertising includes advertising around sports games or for a sports betting product. For example, generic gambling brand logos on football shirts or social media advertisements for horse racing products. Behaviour encompasses all types of gambling providing it occurs in response to a sports‐related advertisement, as defined above.

Given the rapidly expanding evidence base and policy relevance of this research area, an in‐depth and up‐to‐date review would be valuable for policy stakeholders. A review of the 2005 Gambling Act [24] permitted the continuation of self‐regulation of advertising, including sport sponsorship, which was left to the discretion of sporting bodies. Current self‐regulatory policies permit advertising around live sports games [25]. Understanding the evidence for the impact of sports‐related gambling advertising on gambling behaviour is vital to understanding how such policy decisions impact gambling behaviour and subsequent gambling harms. In particular, the use of quantitative evidence in public health economic models, such as those used for alcohol [26], can help to forecast the impact of policy decisions on behavioural and health outcomes and associated costs.

### Aim

This review aims to provide the most systematic and up‐to‐date review of the quantitative literature on sports‐related gambling advertising and its association with gambling behaviour.

## METHODS

### Search strategy

A systematic literature search of studies measuring the direct association between sports‐related gambling advertising and gambling behaviour was undertaken following Preferred Reporting Items for Systematic reviews and Meta‐Analyses (PRISMA) guidelines. The protocol and any amendments can be found on Prospero (CRD42024509195). Following preliminary searches several keywords were identified: (Gambl* OR bet OR wager OR stake) AND (advert* OR ad OR market* or promot* OR sponsor*) AND (sport* OR foot* OR soccer OR AFL OR rugby OR cricket OR racing OR horse OR boxing) AND behavio*. The full search was completed on 13 February 2024 using several research databases: Ovid (Medline, Scopus, PsychInfo, Web of Science, CINAHL) and The Cochrane Library. Supplementary searches were undertaken: citation, author and web‐site searches, in addition to any other studies known to the lead author. The methods of grey literature searching align with those recommended in the literature, specifically using Scopus and web‐sites of relevant organisations and funding bodies [21, 22]. Title and abstracts were inspected by the lead reviewer in Microsoft Excel twice. Two additional reviewers (S.G. and E.C.M.) screened a random 20% of search results (a total of 40%) and any queries were discussed until agreement was reached.

#### Inclusion criteria

The inclusion criteria were: (1) quantitative studies; (2) looking at the relationship between sports‐related gambling advertising and gambling behaviour; (3) related to any sport; (4) in any population; (5) in any country; (6) published up to 13 February 2024; and (7) in the English language or with an English language translation.

#### Exclusion criteria

Exclusion criteria included: (1) qualitative studies; (2) literature or systematic reviews; (3) studies focussed on non‐sports‐related advertising; (4) studies that looked at indirect (mediating) effects or mechanisms of effects; (5) content or frequency analysis studies; (6) studies focusing on safer gambling advertising; and (7) studies not in the English language or with no English language translation. The authors acknowledge the valuable contribution of qualitative research in this area. However, qualitative literature was excluded from this review given it primarily focuses on underpinning mechanisms (such as the reasons why behaviour might change in response to advertising). The current review was interested in the direct behavioural impact of advertising and not why it occurs. Articles looking at safer gambling messaging were excluded because this was perceived to be a separate research question.

#### Advertising definition

This review defined sports‐related gambling advertising as any form of advertising by gambling companies if present during, or related to, any sport game or sports betting product. As such, the content of advertising may expand beyond sports products if it occurs in, or around, sports (e.g. on television during live sports). A bottom‐up coding framework was created using the available data from the review, and results presented based on this framework:

1. Traditional (e.g. television (TV), radio, print)
2. Digital (e.g. on‐line, social media)
3. Embedded (e.g sponsorship, pitch‐side)
4. Direct (e.g. emails or text messages)
5. Inducements (e.g. free bets, stake‐back offers)
6. Aggregated advertising or inducements (e.g. one measure of total frequency of exposure to all types of advertising)

#### Gambling behaviour definition

We defined gambling behaviour as any actual gambling, intent to gamble or urge to gamble, including self‐reported measures. This is not controversial given that both urges or cravings and intentions to gamble are reliably associated with gambling behaviour [26, 27, 28, 29]. We also included self‐perceived impacts of advertising on gambling behaviour, but did not include other measures of the affective response to advertising (aside from urge or craving), because these are not necessarily associated with the desire to enact the behaviour.

### Data synthesis

Measures used varied substantially. This somewhat reflects the difficulty in measuring the behavioural impact of advertising, and the lack of standardised measures of gambling behaviour. There is no agreed measure of a ‘unit’ of gambling like alcohol or smoking. Therefore, a meta‐analysis was not appropriate. The considerable diversity of measures could not be overcome by simply converting effect sizes. Instead, a narrative synthesis was undertaken in which results were grouped by advertising type. A narrative summary of the included studies is provided in Table 1. The characteristics of included studies are detailed in Table 2.

**TABLE 1:** Broad narrative summary of included studies.

No.	Authors	Year	Study type (MMAT)	Advertising type	Summary	Strengths	Limitations
1	Houghton and Moss [38]	2020	Experimental	Digital	This article uses an experimental design to measure individual likelihood of betting, expenditure and confidence in bets presented on social media. Results show that individuals are more likely to bet on certain types of bets (medium complexity) when presented on an affiliate account. This raises concerns about risks of affiliate marketing.	Uses an experimental design and randomises the presentation of advertisements to reduce bias. Looks at social media and affiliate marketing that is under‐researched.	The study may lack external validity. The authors impute missing data, but do not discuss testing the missing data to see if there is any potential bias.
2	Noble *et al*. [49]	2022	Observational	Aggregate	This article exploits a large cross‐sectional survey on adolescent behaviour to estimate the impact of gambling advertising on gambling behaviour in this subgroup. Results indicate that those who are more exposed to sports advertising have higher involvement in gambling in the past 30 days, and are more likely to be an at‐risk or ‘problem’ gambler. However, these results are no longer significant after controlling for a number of confounding variables.	This study uses a large, weighted sample of rich data on youth gambling behaviour with well‐validated measures.	The measure of gambling advertising may fail to capture that sports advertisements can also occur on‐line, and therefore, may still have an effect.
3	Roderique‐Davies *et al*. [43]	2020	Experimental	Embedded	This article uses a randomised pilot experiment to test the impact of embedded gambling advertising on gambling urge and to compare whether this differs between sports (higher risk) and non‐sports (lower risk) students. Participants reported increased urge to gamble when presented with embedded advertising, and this was significantly higher for sports students who had higher PGSI scores. Sports students were also urged to gamble when presented with a sports non‐advertising condition, indicating that there may be an innate association between football and gambling for this subgroup.	This study uses novel experimental methods in this area of research to try and elicit urge to gamble following exposure to embedded advertising.	This is only a pilot study so the sample is small. The experiment may lack external validity because the clips were shorter than a full football game, and were not live.
4	Russell *et al*. [52]	2019	Observational	Aggregate	This study uses a cross‐sectional survey of Australian adults to explore who bets on micro‐events, and which variables are associated with micro‐betting. Results indicate that higher exposure to gambling advertising is associated with reduced betting on micro‐events.	The study uses a large sample and controls for a number of important demographic and gambling‐related confounders.	Micro‐betting is technically not legal in Australia, therefore, it is unlikely that the advertising these people are exposed to is advertising micro‐bets.
5	Hing *et al*. [34]	2019	Observational	Traditional, on‐line, direct, embedded	This study uses EMA methods to measure exposure to, and perceived influence of, wagering advertisements and inducements. Results indicate that all types of advertisements and inducements were more likely to prompt a higher frequency of bets and larger bets among race bettors reporting any influence. For sports bettors, this was true for the frequency of bets. Sports bettors reported placing safer bets in response to advertisements, which might be because of the content of the advertisement decreasing the perceived risk of the bet.	The use of EMA helps to minimise recall bias in advertising exposure while enhancing the ecological validity of findings.	The study uses a non‐representative sample and is mostly descriptive.
6	Hing *et al*. [53]	2018	Observational	Aggregate	Using a cross‐sectional on‐line survey the authors examine the effect of wagering advertisements on ‘impulse’ betting on sport. Results indicate that certain inducements, such as bonus bets, increased ‘impulse’ betting during the game. However, exposure to advertising had a negative association with ‘impulse’ betting during the game. Reporting higher watching of sports was associated with greater ‘impulse’ betting.	This study uses a large sample of data and multiple regression analysis controlling for a number of potential confounding variables.	The authors also rely on the participant's subjective judgement of an ‘impulse’ bet.
7	Russell *et al*. [47]	2018	Observational	Direct, inducements	This study uses EMA to survey race and sports bettors over 1 week of key sporting events in Australia. Participants were asked to forward direct emails and text messages received to the authors' over the same period. Regression models showed that direct messaging was significantly associated with higher intended and actual expenditure on betting. Text messaging was seen as the most important method for actual expenditure, potentially because of its quicker response rate compared to emails.	EMA may reduce recall bias while enhancing the ecological validity of findings. The study asks participants to directly forward emails and texts. It uses regression models that control for individual variability in betting.	The percentage of direct messages forwarded to the researchers was variable and quite low for sports bettors.
8	Hing *et al*. [45]	2017	Observational	Sponsorship, aggregate	This study explored the impact of gambling promotions on sports betting behaviour in a sample of internet sports bettors in Australia. Regression models suggest that exposure to promotions is not significantly associated with ‘problem’ gambling scores, but a higher perceived impact of promotions on behaviour is positively and significantly associated with PGSI scores.	This study uses regression models with controls for important confounders (age, gender, attitudes and approval of promotions).	The authors use a proxy measure of advertising exposure.
9	Hing *et al*. [51]	2016	Observational	Aggregate	This study uses descriptive methods to analyse the impact of demographic, behavioural and normative risk factors for gambling problems. It uses self‐reported watching of sports as a proxy measure for exposure to advertising and finds that increased exposure is associated with a significant increase in total PGSI score.	The study uses a large sample and examines a variety of risk factors for gambling problems.	The authors' do not use regression analysis and, therefore, do not control for potential confounding factors.
10	Di Censo *et al*. [39]	2023	Experimental	Digital, inducements	This study looks at the impact of betting inducements on the perceived betting behaviour of young people in the UK, Australia and New Zealand. Results show that higher‐risk gamblers are more likely to be influenced by inducements, and sign‐up offers are the most influential. When controlling for other confounding factors, regressions indicate that those at a higher risk of harm are more likely to believe that inducements exacerbate their gambling.	This study uses professionally generated advertisements to enhance the external validity of findings and minimise branding effects. It randomly exposed participants to advertisements to reduce order effects. It also undertakes quantitative research in a subgroup where there has been mostly qualitative work.	The study may lack contextual factors because the advertisements were not related to a real‐world live sporting event. They only measure perceived impact on betting, which may differ from actual betting.
11	Hing *et al*. [42]	2015a	Observational	Embedded	This study estimates the impact of exposure to gambling promotions on gambling intentions. Results indicate that higher exposure is associated with increased intentions to bet in the next 6 months, after controlling for additional gambling characteristics. Summary data suggests that individuals with a higher PGSI group report a higher impact of advertising on their frequency, expenditure and time spent betting.	This study uses a panel to recruit participants, which results in a more representative sample with lower risk of missing data.	This study relies on a proxy measure of advertising. The study measures intentions and not actual betting.
12	Hing *et al*. [42]	2015b	Observational	Embedded	This study looks at the average perceived impact of gambling advertising on sports betting behaviour among different PGSI groups. Results indicate that ‘problem’ gamblers report that advertising impacts their frequency, expenditure and time spent betting, whereas other PGSI groups do not. The difference between groups is statistically significant.	This study uses a panel to recruit participants, which results in a more representative sample with low risk of missing data.	This study reports descriptive statistics only.
13	Lopez‐Gonzalez and Griffiths [50]	2021	Observational	Aggregate	This study explores the differing impact of gambling advertising on gambling behaviour between different PGSI groups in a sample of Spanish sports bettors. Results indicate that higher‐risk gamblers report a significantly greater perceived impact of advertising on behaviour compared to lower‐risk gamblers.	This study uses a panel to recruit participants, which reduces biases from missing data or non‐response. It also applies this research question to the Spanish context, which is the only article in this review to do so.	The study measures perceived impact on betting, and not actual impact.
14	Johnston and Bourgeois [44]	2015	Observational	Sponsorship	This study uses hierarchical regression models to explore the existence of a ‘third‐person effect’ in gambling sponsorship advertising. Within their models, the authors' identify that increased exposure to sponsorship advertising is associated with an increased intention to use that sponsor. Additionally, those who perceive sponsorship advertising as having a ‘powerful’ effect on themselves have significantly higher intentions to use the sponsor.	This study uses a potentially more representative quota sample. The authors' also control for a number of potential confounding factors in their models.	The purpose of this study was not to measure the impact of advertising on behaviour.
15	Hing *et al*. [46]	2014	Observational	Sponsorship, aggregate	This study measures the impact of exposure to advertising during sports on the sports betting intentions of young people age 12 to 17 in Australia. Correlation analyses show a significant positive association between exposure to advertising and intention to bet once 18. However, this is no longer significant when included in a regression model with additional controls.	This study is one of few to look at this research question among under 18 year olds. The authors' use an on‐line panel to collect the sample to attempt to make it more representative of the population of interest.	The results might reflect that this is a more general sample rather than a sample who are highly involved in sports watching. The study relies on a proxy measure of exposure.
16	Wardle *et al*. [40]	2022	Observational	Direct, digital	This study explores the impact of gambling marketing on unplanned gambling spend in a large sample of British sports bettors. Results indicate that those at a higher risk of gambling harm are significantly more likely to report that gambling marketing prompts unplanned gambling spend compared to those at no risk of harm. The effect sizes are large, particularly for ‘problem’ gamblers. Additionally, exposure to direct marketing and to a gambling brand on social media increase the likelihood of reporting that marketing prompts unplanned spend.	This study uses a large and weighted, therefore, representative sample of British sports bettors. The authors' control for a number of important confounding variables in their regression models.	The study cannot measure the value of unplanned spend.
17	Browne *et al*. [69]	2019	Observational	Direct, TV, inducements, aggregate	This article uses EMA methods to estimate the impact of exposure to advertisements and inducements on intended, actual and excess gambling spend among race and sports bettors. The results indicate that aggregate exposure to advertisements and inducements is significantly associated with likelihood of betting and actual spend on betting. For race bettors only, this association applies to excess spend too. Specific inducements that have an impact are stake‐back offers, direct messaging and TV advertising.	This study uses EMA methods, which help to enhance the ecological validity of data and minimise response biases. The authors are also able to collect actual betting spend data.	There was significant attrition in the surveys.
18	Rockloff *et al*. [48]	2019	Experimental	Inducements	This study uses an on‐line experiment to explore the impact of inducement type on selection of odds (short, medium, long). Results indicate that participants were significantly more likely to select longer odds when presented with inducements versus no inducements. Only cash rebate showed an independent significant effect. No differences were found by PGSI group.	This study uses an experiment that has strong internal validity because the researchers can control for exposure to inducements and can minimise contextual confounding factors.	This study may lack external validity because it lacks these contextual factors that may impact betting behaviour (e.g. betting on a live game).
19	Sproston *et al*. [41]	2015	Observational	Traditional, digital	This report explores the impact of sports and race betting marketing on gambling behaviours in a large sample of adults and adolescents. Logistic regression models indicate a strong positive correlation between exposure to digital sports betting marketing and sports betting in the last 12 months. Betting on racing in the last 12 months was significantly associated with exposure to traditional (TV and radio) race betting marketing. Higher frequency of betting on EGMs and other gambling types was associated with increased exposure to traditional race marketing, and also digital race marketing for the latter. In the adolescent sample, only exposure to digital race marketing was associated with likelihood of betting on other activities.	This report uses a large sample of adults and adolescents (who are rarely researched in this area).	The adolescent sample size is small.
20	Schottler Consulting [35]	2012	Observational	Aggregate	This report explored the impact of sports gambling marketing on unplanned betting and unplanned gambling spend in a sample of Australian adults. Descriptive results showed that on average, participants did not feel that sports betting marketing prompted them to spend money or bet when they had not intended to. However, risk of ‘problem’ gambling was a significant predictor of influence of marketing on unplanned behaviour in a stepwise regression, although the correlations were low.	This report uses a weighted sample for the population of bettors in New Zealand.	The results of this study are descriptive. It may have been difficult for respondents to categorise planned vs. unplanned behaviour that may lead to some reporting biases.
21	Russell and Hing	2020	Observational	Aggregate	This report investigates advertising before and during the initial COVID‐19 lockdown period in Australia. Descriptive results indicate that, on average, respondents felt that advertising did not impact their gambling expenditure in either period. However, participants were significantly more likely to report a reduction in expenditure because of advertising during lockdown compared to before, which was a period where exposure to advertising was lower. This may indicate a small protective effect of reduced advertising.	This report uses a large sample of respondents to explore an interesting period where sports betting advertising reduced because of the halting of live sports.	Results are descriptive. A large percentage of the sample had not bet at all, had not bet on sports and held no accounts with operators in the 12 months before lockdown.
22	Jenkinson *et al*. [37]	2023	Observational	Traditional, embedded, digital, aggregate	This report explored the impact of sports and race betting advertising on gambling behaviours in Australia. Results indicated a strong correlation between exposure to advertising and betting on sports or racing. Additionally, 20%–30% of participants reported that any type of advertising influenced the amount they bet, whether they bet on impulse, whether they tried a new product and whether they started betting at all. These results were more pronounced in younger and at‐risk individuals.	This report uses a large sample, which is representative in terms of age, gender and location of residence.	The results are descriptive.

Abbreviations: COVID‐19, coronavirus disease 2019; EGMs, electronic gaming machines; EMA, Ecological Momentary Assessment; MMAT, Mixed Methods Appraisal Tool; PGSI, Problem Gambling Severity Index; TV, television; UK, United Kingdom.

**TABLE 2:** Summary of study characteristics and results.

Study characteristics								
No.	Funding	Setting	Population	*N*	Sampling method	Definition of bettors	Methods	Statistical methods
1	Gamble Aware	UK	18+	100	Opportunity	Regular football bettors (at least once a month)	Experimental	Two‐way factorial ANOVA and independent sample *t* tests
2	Victorian Responsible Gambling Foundation	Australia	12–17	4993–6377	Random (weighted)	N/A	Cross‐sectional survey	Logistic mixed regression models (with controls)
3	No mention of funding	UK	18 + (students)	60	Opportunity	Gambled at least once in the last 12 months (including National Lottery)	Randomised Experimental	Two‐way factorial ANOVA and independent sample *t* tests
4	Centre for Gambling Education and Research (Southern Cross University)	Australia	18+	1813	Convenience	Bet on sports in the last 12 months	Cross‐sectional survey	Two‐step zero inflated regression (with controls)
5	Victorian Responsible Gambling Foundation	Australia	18+	722	Convenience	Bet on horse/greyhound racing or sports betting at least fortnightly	Ecological Momentary Assessment (longitudinal)	Descriptive statistics (%)
6	Victorian Responsible Gambling Foundation	Australia	18+	1813	Convenience	Bet on sports in the last 12 months	Cross‐sectional survey	One‐way ANOVA and multiple linear regression (with controls)
7	Queensland Department of Justice and Attorney General (Responsible Gambling Research Grant)	Australia	18+	455	Purposive	More than 0% betting via the internet	Cross‐sectional survey	Zero‐inflated regression models (with controls)
8	Victorian Responsible Gambling Foundation	Australia	18+	202	Convenience	At least fortnightly race or sports bettors	Ecological Momentary Assessment (longitudinal)	Negative binomial regression (with controls)
9	Queensland Department of Justice and Attorney General (Responsible Gambling Research Grant)	Australia	18+	639	Purposive	Bet on sports in the last 12 months	Cross‐sectional survey	Spearman's correlation and Kruskal‐Wallis tests
10	NSW Office of Responsible Gambling (NSW Responsible Gambling Fund)	UK/Australia/New Zealand	18–24	130	Purposive	Prior experience with sports betting	Cross‐sectional survey	Two (risk‐level) by four (inducement type) mixed ANOVAs and hierarchical regression models (with controls)
11	Queensland Department of Justice and Attorney General (Responsible Gambling Research Grant)	Australia	18+	1000	Purposive	N/A	Cross‐sectional survey	Summary statistics and hierarchical regression (with controls)
12	Queensland Department of Justice and Attorney General (Responsible Gambling Research Grant)	Australia	18+	544	Purposive	At least fortnightly sports bettors	Cross‐sectional survey	Summary statistics (mean values) and ANOVA
13	Grant from the Government of the Basque Country, Spain and Spanish Organisation of the Blind	Spain	18+	659	Purposive	Bet on sports in the last 12 months	Cross‐sectional survey	Kruskal‐Wallis and χ^2^ tests
14	University of Queensland Postdoctoral Research Fellowship	Australia	18+	511	Quota	N/A	Cross‐sectional survey	Hierarchical regression (with controls)
15	Queensland Department of Justice and Attorney General (Responsible Gambling Research Grant)	Australia	12–17	131	Purposive	N/A	Cross‐sectional survey	Hierarchical regression (with controls)
16	The Economic and Social Research Council/The Wellcome Trust	UK	18+	3195	Purposive (weighted)	Bet at least monthly on sports	Cross‐sectional survey	Logistic regression (with controls)
17	Victorian Responsible Gambling Foundation	Australia	18+	597	Purposive	At least fortnightly sports bettors	Ecological Momentary Assessment (longitudinal)	Linear mixed effects regression models (with controls)
18	Victorian Responsible Gambling Foundation	Australia	18+	299	Purposive	Bet on AFL, cricket, or soccer at least twice in the previous 12 months	Experimental	Wilcoxon signed rank test, ANOVA, χ^2^ test
19	Gambling Research Australia	Australia	13+	3200	Purposive	Gambling at least once a month	Cross‐sectional survey	Logistic regression (with controls)
20	New Zealand Ministry of Health	New Zealand	18+	157	Quota (weighted)	Gambled at least once in the last 12 months (including Lotto)	Cross‐sectional survey	Summary statistics (mean Likert) and stepwise regression (with controls)
21	Victorian Responsible Gambling Foundation	Australia	18+	2120	Purposive	Gambled at least once in the 12 months before initial lockdown, or in the 2 months of lockdown	Cross‐sectional survey	Summary statistics (% Likert, McNewmar‐Bowker test)
22	Australian Gambling Research Centre	Australia	18+	1765	Community (aligned with population parameters)	N/A	Cross‐sectional survey	Summary statistics (% Likert)

Abbreviations: AFL, Australian Football League; ANOVA, analysis of variance; NSW, New South Wales; UK, United Kingdom; N/A, Not Applicable.

### Assessment of study quality

Study quality was assessed using the Mixed‐Methods Appraisal Tool (MMAT). The MMAT was developed in 2006 [30], and subsequently revised [31]. It allows for the appraisal of studies in five categories of study type: (1) qualitative; (2) quantitative randomised‐controlled; (3) quantitative non‐randomised; (4) quantitative descriptive; and (5) mixed‐methods. The MMAT was selected because it allows for assessment of descriptive studies. Quality criteria in the MMAT cover: reporting (research questions and aims), sampling (strategy, representativeness), measures (appropriateness, validity), data (suitability) and statistical methods (confounding and risk of bias). The MMAT does not have an associated scoring system and as such studies cannot be ranked or compared based on their quality level. Instead, the tool encourages the inclusion of all articles, regardless of quality, to allow for a detailed presentation of the strengths and weaknesses of the evidence base. An explanation of the type of studies included in this review is provided in Appendix A.

## RESULTS

### Search results

**FIGURE 1:**
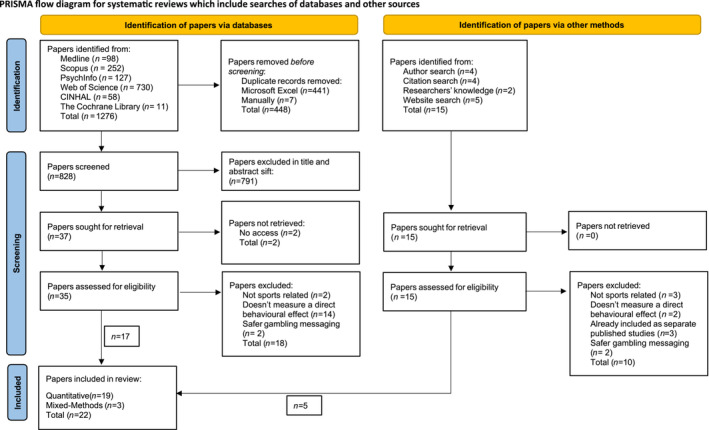
Preferred Reporting Items for Systematic reviews and Meta‐Analyses (PRISMA) flow diagram for systematic reviews.

Figure 1 presents a PRISMA flow diagram citing the reasons for excluding studies at each stage. In total, 1276 articles were identified during the full systematic search of databases and exported from Zotero into Microsoft Excel. Of these, 441 duplicates were deleted using Microsoft Excel, and a further seven duplicates were deleted manually. Following the title and abstract sift, 791 articles were excluded. Of the remaining 37 studies, 35 were extracted successfully by the lead reviewer (E.M.) and two were excluded as they were not accessible [32, 33]. Four articles were identified from citation searches of included studies. Searches of authors who appeared more than once in the author list of included studies identified an additional five articles. Further search strategies included web‐sites of relevant gambling and government organisations (five articles identified) and the lead reviewer's knowledge (two articles identified). See Appendix B for a list of authors and web‐sites searched. After the full‐text search, a total of 22 studies were included in the final review.

### Description of included studies

#### Characteristics

The broad methodologies of included studies were experimental (*n* = 4), and observational (*n* = 18) (Table 1). The majority of studies used cross‐sectional surveys (*n* = 15), with a small number of longitudinal studies (*n* = 3). A substantial number used measures of perceived impact of advertising on gambling behaviour (*n* = 10). For a description of the measures used in each specific study, see Appendix C.

Studies took place in Australia (*n* = 16), the United Kingdom (UK) (*n* = 3), Spain (*n* = 1), New Zealand (*n* = 1) and a combination of these countries (*n* = 1). Sampling methods included purposive (*n* = 12), convenience (*n* = 4), quota/stratified (*n* = 3), opportunity (*n* = 2) and random weighted sampling (*n* = 1). Samples comprised adults (*n* = 19), adolescents (*n* = 2) and mixture of both (*n* = 1). The majority of studies were funded by independent government organisations (*n* = 18). Other funding sources included independent trusts/charities (*n* = 1), postdoctoral research fellowships (*n* = 1) and non‐independent gambling charities (*n* = 1). One study did not report any funding source. A more detailed data extraction table is available in Appendix D.

## RESULTS

### Traditional (e.g. TV, radio, print)

Several studies explored the impact of traditional marketing on gambling behaviour. One study [34] reported that 29% to 43% of sports bettors and 30% to 38% of race bettors reported ever feeling influenced by television, radio or print advertisements. This was higher than on‐line advertising, but lower than embedded and direct forms. A grey literature report found that a higher frequency of race betting, and frequency of using electronic gaming machines (EGMs), was significantly associated with higher exposure to traditional race betting marketing [35]. There is some evidence that TV advertisements influence actual expenditure on gambling for race bettors in Australia [36]. An Australian study also reported that television advertising was the most influential advertising media for initiating betting (1 of 7 people) and changing what people bet on (1 of 10 people) [37].

### Digital (e.g. web‐sites, social media)

Approximately 30% to 50% of sports bettors, and 27% to 47% of race bettors report ever being influenced by on‐line advertising on web‐sites or social media in an Australian study [34]. Two studies used fake social media advertisements, constructed by the researchers, to measure bet complexity [38] and gambling risk level using the Problem Gambling Severity Index (PGSI) [39]. Researchers exposed individuals to advertisements and measured responses. Participants increased the complexity of bets when an advertisement was present on an affiliate (partner) social media account compared to an operator. This was despite being significantly more likely to place lower complexity bets and spend less on higher complexity bets, overall [38]. Additionally, higher‐risk gamblers (measured using a 10‐item subscale of high‐risk gambling developed using diagnostic and symptomatic criteria) were more likely to believe that inducements on social media exacerbate their gambling harms [39]. Being exposed to marketing on social media on at least one platform significantly increased the likelihood of unplanned gambling spend in a sample of sports bettors [40]. A report [41] indicated that higher exposure to sports betting marketing through digital media was associated with gambling on sports in the last 12 months. Additionally, a higher frequency of gambling on other types of activities was significantly associated with increased exposure to digital race betting marketing. In the adolescent sample, only digital race betting marketing was significantly associated with higher intention to gamble on other activities.

### Embedded (e.g. sponsorship, pitch‐side)

Various studies explored embedded advertising within sports broadcasts. One study suggested that approximately 40% of sports and race bettors report ever being influenced by embedded advertising [34]. Another [42] suggested that higher‐risk gamblers report a significantly higher impact of embedded gambling advertising during televised sport on their perceived frequency, expenditure and time spent betting on sports, although overall scores were low. One study indicated that direct advertising had the highest impact on increased betting (17% reported this impact) and placing ‘impulse’ bets (13% reported this impact) [37]. One study used randomised methods to estimate the impact of embedded advertising on gambling urge [43]. When exposed to a professional football game with embedded gambling advertising, students reported higher urges to gamble, and urges were significantly higher for sports compared to non‐sports students. Sports students were perceived to be higher‐risk because of their comparatively higher mean PGSI scores. Another study [42] revealed that exposure to this type of advertising was associated with an increased intention to bet in the next 6 months. An Ecological Momentary Assessment (EMA) study in Australia suggested that brand promotion influenced actual bet spend among sports bettors [36]. EMA involves surveying participants’ behaviour in a natural environment. In this context, it involved measuring self‐reported exposure to advertising and gambling behaviour as close as possible to their actual occurrence. One study [44] found that increased exposure to sponsorship advertising was significantly associated with intentions to use a sponsor. Additionally, adults who perceived sponsorship advertising to have a ‘powerful’ effect on themselves exhibited a higher intention to use that sponsor. Another article [45] reported that individuals with a higher PGSI score, and therefore, at a higher risk of harm, were more likely to report that they would use gambling products in response to gambling sponsorship. However, in an adolescent sample, there was a general disagreement that sponsorship would lead to the use of a sponsor's product [46].

### Direct (e.g. emails or texts)

One study indicated that 57% of sports bettors and 24% of race bettors reported ever being influenced by direct messaging [34]. Individuals experiencing harm from gambling are significantly more likely to report that direct marketing had prompted unplanned gambling spend compared to those not experiencing harm. Being exposed to at least one or more types of direct marketing also significantly increased the likelihood of unplanned gambling spend in the same study [40]. Another study reported that the total number of direct messages received was associated with an increased likelihood of betting for race and sports bettors and an increased amount bet for race bettors [47]. Direct advertising through text messages increased actual betting in the whole sample. The number of direct emails was associated with an increased intention to bet, and intention to bet with larger amounts, for the whole sample, and the likelihood of actually placing a bet for sports bettors only. Direct messaging is reported to influence intended, actual and excess spend among Australian race bettors [36].

### Inducements (e.g. free bets or stake‐back offers)

One study used simulated videos of sports games to measure the effect of inducements on riskiness of betting [48]. Participants chose longer, riskier, odds when inducements were present during a sports game compared to when they were not. Cash inducements were particularly influential, exhibiting a greater risk profile [48]. In another study [39], social media advertisements with a sign‐up inducement were best at explaining higher risk gambling scores in men age 18 to 45 compared to all other inducement types. An EMA study in Australia indicated that stake‐back offers influenced actual bet spend for race bettors [36]. In models without additional controls, the number of direct messages containing stake refund offers and bonus odds were associated with actually placing a bet, and sign‐up and match‐stake inducements increased the amount bet among race bettors [47]. For sports bettors, these results were less clear with bonus wins and direct messages with no inducements increasing the likelihood of placing a bet in models without controls. Stake‐back, multi‐bet and match‐your‐stake inducements were particularly influential in one study [34].

### Aggregate (total frequency of exposure across all types)

In an Australian EMA study, aggregate exposure to advertising and inducements increased actual expenditure on gambling [36]. Aggregate exposure to advertising also increased excess spend among race bettors. Two studies surveyed adolescents and found that overall exposure to sports gambling advertising was associated with increased intentions to gamble when they reached the legal age [46], likelihood of gambling in the last 30 days, gambling on ‘hard’ activities (e.g. casino games) and being a higher‐risk gambler [as measured using the Diagnostic Statistical Manual IV adapted for juveniles (DSM‐IV‐[MR]‐J)] [49]. However, these relationships were not statistically significant when other control variables were included in the model.

Higher‐risk gamblers reported that advertising influences their frequency, expenditure and time spent betting to a greater degree than lower‐risk gamblers in one study [42]. Higher‐risk gamblers report a greater impact of advertising on frequency of betting on sports compared to lower‐risk gamblers in another [50]. Watching more live sports, and therefore, being more frequently exposed to gambling advertising, is reportedly associated with a higher PGSI score [51]. Other studies support these findings, but results were not statistically significant [45].

Studies looking at ‘micro’ or ‘impulse’ betting found evidence contradicting the other studies in this review [52, 53]. Betting on micro‐events (a form of in‐play betting) is controversial because it reduces the time between betting and the outcome and, therefore, may be more harmful. These are similar to ‘impulse’ bets defined by Hing and colleagues [53] as spontaneous or unplanned bets. These studies found that exposure to advertising were associated with a reduced likelihood of placing these types of bets.

A report in New Zealand found, on average, a very low mean likelihood of placing a bet or unplanned spending on gambling in response to advertising [35]. However, descriptive results from an Australian report found that younger people and higher‐risk individuals were more likely to report that gambling advertising influenced the amount they bet, whether they bet on impulse, whether they tried a new product or whether they started betting for the first time [37]. There was also a strong association between exposure to advertising and betting on sports or racing in the previous 12 months. Finally, a report [54] described weak‐to‐moderate positive correlations between frequency of exposure to advertising of sports and race betting and frequency of gambling on each form. Correlations for sports betting decreased during lockdown, and respondents were more likely to report that expenditure had decreased because of advertisements during this time. This may have been a period of reduced advertising because of the sports shutdown in Australia, therefore, it may suggest a small protective effect of this reduced advertising on gambling behaviour, which is further supported by respondents reporting less frequent exposure to sports advertising during lockdown [54].

### Quality of methodologies

Overall, studies reported their aim(s) or research question(s) clearly within the article and they collected data that was suitable for answering their research question. However, most measures of advertising exposure were proxied measures. The main limitation of this evidence is that most studies fall under the observational category, and therefore, are less able to establish causal relationships. Data tends to be self‐reported cross‐sectional and may be subject to bias. Samples tend to be non‐representative. However, this is appropriate for the context because studies over‐recruit higher‐risk gamblers to ensure that there are sufficient numbers in each gambling risk category. Additionally, research questions often mean that purposive or convenience samples of regular gamblers are necessary. The use of panels to recruit samples might introduce some bias given these people have signed up to take surveys, so they may differ somewhat from the general population [55]. A limitation of this evidence base is the lack of standardised measures of advertising or gambling behaviour. However, there is no agreed measure of a unit of gambling behaviour, and measuring advertising exposure is difficult outside of controlled experiments.

Despite this, the use of experimental methods in recent years enhances the internal validity of findings because researchers can control for actual exposure to advertising. Other promising studies use EMA, which reduce recall biases and enhances the external validity of findings [56] by measuring exposure to advertising and gambling behaviour as close to their occurrence as possible and in a real‐world setting. Despite their drawbacks, using on‐line panels to recruit samples can enhance the completeness and quality of data collected. More recent studies use large, weighted population samples of bettors, which improves representativeness of samples. Several studies use regression models with controls for potential confounding factors (such as age, sex and previous gambling behaviour). For a more detailed examination of the methodological quality of each study, see Appendix E.

## DISCUSSION

The review aimed to provide the most systematic and up‐to‐date review of the quantitative literature on sports‐related gambling advertising, as defined in this study, and its relationship with gambling behaviour. We narratively synthesised and critically analysed the evidence to identify knowledge gaps using refined search criteria to answer a research question of relevance to public health policy. The evidence suggests that there is a positive association between sports‐related gambling advertising and gambling behaviour. Descriptive results indicate that this may be more pronounced in higher‐risk gamblers who are already at increased risk of harm. These results hold across different advertising media.

Young adults at higher risk of gambling harm may be more affected by embedded advertising during sports, and within this group, watching football may go hand‐in‐hand with sports betting. Furthermore, sponsorship advertising might increase the likelihood of using a sponsor's products among adults. There is preliminary evidence for an association between sports‐related advertising and gambling behaviour in samples under the age of 18, although results were not always statistically significant. This could be partly because of small sample sizes. Social media advertising via affiliate accounts may contribute to gambling harm by increasing the complexity of bets placed because of their differential framing of bets as lower risk. Inducements that reduce the risk or cost of gambling, such as sign‐up or stake‐back offers, might have a greater impact. Direct messaging, especially texts because of their quicker response time might be an important influence on gambling behaviour. The self‐reported impact of advertising on behaviour appears to be more pronounced among higher‐risk gamblers, as measured using the PGSI. The contradicting effects found in studies on in‐play betting might occur because this type of betting is not technically legal (micro‐betting) or as easily accessible (in‐play) in the country studied (Australia). These results corroborate and supplement the results found in previous reviews [15, 16, 17].

Future research should prioritise experimental and longitudinal studies to strengthen the evidence base. Randomised experimental studies, in which people are randomised to advertising exposure, are required to demonstrate causal inference. There are some examples of these in this review. Quasi‐experimental studies, where external variation in advertising exposure is used to infer causality, would be useful for enhancing the ecological validity of findings. Collecting actual betting data from individual accounts could reduce the risk of reporting bias. Studies on adolescents should focus on obtaining larger samples. There will likely be improvements in this area of research as we collect more data on gambling behaviour. This year, one of the largest survey on gambling behaviour in the world commenced in Great Britain [57]. Such datasets broaden opportunities for future research.

### Strengths and limitations

This review provides the most up‐to‐date and systematic review of the literature on the relationship between sports‐related gambling advertising and gambling behaviour. This is relevant to current gambling policy in the United Kingom and world‐wide. It also refines the search criteria of previous reviews to look at quantitative evidence. This makes the results of this review more relevant for health economic modelling, which can be used to measure the impact of policies on behaviour and subsequent health.

A single quantitative effect size for sports‐related advertising, which could be used in health economic modelling, could not be defined at this stage because a meta‐analysis was not appropriate. This review excluded studies not published in English and most of the studies were conducted in Australia. This restricts our ability to make cross‐country comparisons. The same issue applies to the age range of studies, which mostly cover adult populations. Future research should look at including other countries and age groups. The accuracy of grey literature searching and its potential contributions to systematic reviews is contested. There is a risk that evidence may be missed or be of lower quality because of a lack of peer review. Nonetheless, it was appropriate in this case for reasons provided. There is one study where we cannot definitively say that sports‐related advertising was measured [40], but we make the assumption that it was most likely to be this type of advertising given that it is a sports betting sample.

### Implications for public health policy

The implication of these findings is that current policies that allow for sports‐related gambling advertising may be contributing to gambling harm by increasing betting. Further research is needed to corroborate this and strengthen the case for causal effects and to provide quantitative evidence that can be used in health economic modelling of gambling harms. The acceptance of self‐regulation of gambling advertising in the United Kindgom is justified by the claim that there is no direct causal evidence for advertising's impact on population health [24, 58], because studies only indicate a direct link to participation in gambling and not to health outcomes. However, we can indirectly assume that the increased gambling behaviour reported in these studies is exacerbating harms given the evidence for the relationship between harms and increased expenditure and frequency of betting [59, 60, 61, 62, 63], despite disagreement over the shape of this relationship [64]. Low‐risk gambling guidelines in Canada also encourage reduced spending and frequency of gambling to minimise harm [65]. To strengthen the evidence base, future studies on advertising might include measures of health‐related quality of life to estimate the direct impact of exposure to advertising on population health. Despite the limitations of measures [66], there is some evidence for the impact of gambling on population health‐related quality of life [67, 68, 69]. This requires further research. In the meantime, government intervention may be justified based on the evidence for links between advertising and gambling and gambling activity levels and gambling‐related harm. This is especially important given that this effect may be more pronounced in higher‐risk gamblers who are already more vulnerable to harm. The World Health Organisation recommends comprehensive advertising policies for alcohol and tobacco [70, 71]. It may be time for governments to adopt these recommendations for gambling.

A review gambling legislation in the United Kingdom [24] has not detailed any major changes to gambling advertising. It requests consultations on cross‐selling of products; this could be beneficial given the evidence on direct marketing in this review. There is no statement on restricting inducements, which have been identified as potentially influential in this review, except for encouraging a socially responsible approach. Sport sponsorship has been left to the discretion of sports governing bodies. The Premier League has agreed to a front‐of‐shirt gambling sponsorship ban for the 2026 to 2027 season, but this does not include sponsorship visible on the arms of football shirts or on pitch‐side hoardings. The evidence in this review highlights some potentially influential types of advertising such as text messaging and pricing promotions. Without specific guidance on how these types of advertisements may be used in a way that minimises their negative effects, we may see increased gambling harms.

There are global implications of this research beyond the United Kingdom. In Australia, calls to restrict gambling advertising during live sports are growing since a 2023 parliamentary report recommended a phased total ban on on‐line gambling advertising and sponsorship in the next 3 years [72]. Some European countries have implemented variations of partial to full advertising bans, some self‐regulatory [69, 70, 71, 73, 74, 75]. The success of some of these policies is contested [76, 77]. The findings reported here support the need for intervention using comprehensive approaches, which include all types of advertising media, to protect those most vulnerable to harm.

## CONCLUSION

This systematic review explored the relationship between sports‐related gambling advertising, as defined in this study, and gambling behaviour. It concluded that there is a positive association between different types of sports‐related gambling advertising and gambling behavioural outcomes. This finding may be more pronounced in higher‐risk gamblers, who are at increased risk of harm. Future research should expand on experimental and longitudinal evidence and consider including gambling health‐related quality of life outcome measures, to strengthen the evidence base. In the meantime, governments might intervene based on the precautionary principle and the indirect evidence of gambling harm.

## AUTHOR CONTRIBUTIONS

**Ellen McGrane:** Conceptualization (lead); data curation (lead); formal analysis (lead); funding acquisition (lead); investigation (lead); methodology (lead); project administration (lead); writing—original draft (lead). **Robert Pryce:** Methodology (supporting); project administration (supporting); supervision (supporting); writing—original draft (supporting). **Matt Field:** Methodology (supporting); project administration (supporting); supervision (supporting); writing—original draft (supporting). **Shangshang Gu:** Formal analysis (supporting); methodology (supporting); writing—original draft (supporting). **Esther C. Moore:** Formal analysis (supporting); methodology (supporting); writing—original draft (supporting). **Elizabeth Goyder:** Conceptualization (supporting); formal analysis (supporting); investigation (supporting); methodology (supporting); project administration (supporting); supervision (lead); writing—original draft (supporting).

## DECLARATION OF INTERESTS

E.M. is funded by a Wellcome Trust grant (224 852/Z/21/Z). The authors have no conflicts of interest to declare.

## Supporting information


**APPENDIX A:** STUDY TYPES (MMAT).
**APPENDIX B:** DETAILS OF SUPPLEMENTARY SEARCHES.
**APPENDIX C:** MEASURES USED IN EACH STUDY.
**APPENDIX D:** DETAILED DATA EXTRACTION TABLE.
**APPENDIX E:** DETAILED QUALITY ASSESSMENT TABLE.

## Data Availability

There is no data available to share since this was a systematic literature review.
