# Supplementary material for: What is the impact of sports‐related gambling advertising on gambling behaviour? A systematic review
Source: Addiction. 2025 Jan 11;120(4):589–607. doi: 10.1111/add.16761 (PMC11907332; doi:10.1111/add.16761)
Supplement: Supplementary file 1 — APPENDIX A: STUDY TYPES (MMAT). APPENDIX B: DETAILS OF SUPPLEMENTARY SEARCHES. APPENDIX C: MEASURES USED IN EACH STUDY. APPENDIX D: DETAILED DATA EXTRACTION TABLE. APPENDIX E: DETAILED QUALITY ASSESSMENT TABLE. [file ADD-120-589-s001.pdf]

APPENDIX A: STUDY TYPES (MMAT)

**Study Type (MMAT)**	**Description**
Quantitative randomised	A study in which individuals are allocated to an intervention or control group by randomisation. For the purpose of this review these are called experimental studies.
Quantitative non-randomised	Quantitative studies where the impact of an intervention or exposure (advertising) does not use randomisation to allocate treatment and control groups. For the purpose of this review these studies are combined with the above category (experimental) since the researcher assigns the exposure to participants.
Quantitative descriptive	These are concerned with describing the distribution of variables without specific regard to causal relationships. For the purpose of this review these are called observational studies, and they describe studies where the researcher does not allocate the exposure to advertising but attempts to measure it separately. The mixed-methods (n=3) studies in this review fall into this category based on their quantitative section.

APPENDIX B: DETAILS OF SUPPLEMENTARY SEARCHES

List of Authors Searched	List of Websites Searched
• Alex Russell<br>• Nerilee Hing<br>• En Li<br>• Anna Thomas<br>• Rebecca Jenkinson<br>• Peter Vitartas<br>• Matthew Browne<br>• Matthew Lamont<br>• Elian Fink	• Gamble Aware (research library)<br>• The Gambling Commission (statistics and research)<br>• Gam Care (data and insight/policy and research)<br>• National Problem Gambling Clinic<br>• Gordon Moody Association<br>• Gamblers Anonymous<br>• Open Grey (DANS)<br>• Gam-anon (publications)<br>• Victorian Responsible Gambling Foundation (publications)<br>• Advisory Board for Safer Gambling UK (publications)<br>• Gambling Watch Scotland<br>• Glasgow City Gambling Harms<br>• Citizens Advice Bureau<br>• Australian Gambling Research Centre (research findings)<br>• Gambling Research Exchange Ontario (evidence centre)<br>• International Centre for Youth Gambling Problems and High-Risk Behaviour (research and publications)<br>• AUT Gambling & Addictions Research Centre (our research)<br>• Alberta Gambling Research Institute (publications and statistics)<br>• Responsible Gambling Council<br>• Betting and Gaming Council<br>• Problem Gambling Foundation New Zealand<br>• Gambling Commission New Zealand<br>• Gov.uk (research and statistics)<br>• Department for Culture, Media and Sport (research and statistics)<br>• Australasian Gaming Council (research and policy)

APPENDIX C: MEASURES USED IN EACH STUDY

No.	Authors	Year	Relevant Gambling Behaviour Measure	Behaviour Type	Advertising Measure	Advertising Type
1	Houghton & Moss	2020	Immediate likelihood to bet on a visual analogue scale (0 to 100); Bet stake (£)	Likelihood of betting and amount bet	Participants exposed to fake sports advertising tweets from operator and affiliate accounts in an experimental setting.	Digital
2	Noble et al.	2022	Self-reported ever gambled in the last 30 days (yes/no); types of gambling activities in the last month ('hard' types e.g. casino, card, sports games, poker machines, horse racing and 'soft' types e.g. bingo, lottery, scratch cards); Diagnostic Statistical Manual-IV adapted for Juveniles (DSM-IV-[MR]-J; revised to yes/no response)	Gambling over recall period (30 days), Gambling risk level	Adaptation of Hing et al., (2014) exposure to advertising scale (specifically the awareness of sports/celebrity ads in the previous 30 days)	Aggregate
3	Roderique-Davies et al.	2020	Immediate self-reported urge to gamble using the Gambling Urge Scale (Raylu & Oei, 2004); Problem Gambling Severity Index	Urge to gamble, Gambling risk level	Pre-recorded videos of sports games with and without embedded gambling promotions (plus a non-sports control video)	Embedded
4	Russell et al.	2019	Self-reported percentage of sports bets that the respondent placed on micro events.	Gambling over recall period (30 days)	Estimated using frequency of watching 9 major sports; Reporting how frequently they saw/heard gambling advertisements (Likert; never to almost always)	Aggregate

5	Hing et al.	2019	Perceived influence of past 24-to-48 hour exposure to advertising and inducements on betting (Influence/No influence); How it influenced betting (bet amount, safety, risk); Problem Gambling Severity Index	Perceived influence of advertising on behaviour (24-48 hours recall), Gambling risk level	Whether race/sports betting advertisements and inducements were seen/heard in the previous 24 to 48 hours (Yes/No)	Traditional, Online, Direct, Embedded
6	Hing et al.	2018	Self-reported percentage of past year sports bets that were 'researched and planned in advance', 'on impulse before the start of the match' and 'on impulse during the match'; Problem Gambling Severity Index	Gambling over recall period (1 year)	How frequently they watched 9 major sports in the most recent season; How often they heard/saw advertisements (never, sometimes, most of the time, almost always).	Aggregate
7	Russell et al.	2018	Self-reported actual race and sports betting expenditure; intended race and sports betting expenditure (both over the previous 24 hours).	Gambling over recall period (24 hours), actual and intended	Total number of direct messages received; total number of emails received; total number of texts received; total number of inducements received (all self-reported over previous 24 hours in EMA survey, actual texts and emails forwarded to the researchers)	Direct, Inducements
8	Hing et al.	2017	Problem Gambling Severity Index score	Gambling risk level	Self-reported frequency of watching eight types of televised professional sport (sports where advertising is most prominent) in the most recent season (7-point Likert; never to daily); Sponsorship response scale (Speed & Thomas, 2000)	Sponsorship, Aggregate

					for likely use of sponsors product	
9	Hing et al.	2016	Problem Gambling Severity Index score	Gambling risk level	Self-reported frequency of watching eight types of televised professional sport (sports where advertising is most prominent) in the most recent season (7-point Likert; never to daily)	Aggregate
10	Di Censo et al.	2023	Perceptions of sports betting promotions scale (likelihood of influencing them to bet, and to engage in high risk gambling behaviours)	Perceived influence of advertising on behaviour	Fake social media advertisements for a fictional betting company.	Digital, Inducements
11	Hing et al.	2015a	Self-reported perceived impact of gambling promotions on behaviour (increased frequency of sports betting, caused them to spend more money than intended on sports betting) on 5-point Likert scale (strongly agree to strongly disagree)	Perceived influence of advertising on behaviour	Self-reported frequency of watching eight types of televised professional sport (sports where advertising is most prominent) in the most recent season (7-point Likert; never to daily)	Embedded
12	Hing et al.	2015b	Self-reported perceived likelihood of 11 promotional techniques encouraging them to bet on sport; Whether promotions had increased frequency/time/expenditure on gambling., or caused them to spend more time and money than intended on sports betting, or caused them or someone close to them harm; Problem Gambling Severity Index group	Perceived influence (likelihood) of advertising on behaviour	Self-reported frequency of watching eight types of televised professional sport (sports where advertising is most prominent) in the most recent season (7-point Likert; never to daily)	Embedded

13	Lopez-Gonzalez & Griffiths	2021	Self-reported perceived impact of sports gambling promotions on gambling behaviour from Hing et al., (2015a); Spanish adaptation of the Problem Gambling Severity Index	Perceived influence of advertising on behaviour	No specific measure (just the self-reported variable).	Aggregate
14	Johnston & Bourgeois	2015	Self-reported perceived gambling intentions (e.g. "in the next 12 months if a gambling company sponsors my favourite sport I probably will/will not place a bet with that sponsor....")	Perceived influence of advertising on intentions	Self-reported frequency of exposure to gambling-linked sponsorship advertising (e.g. on players' uniforms) on 5-point Likert scale (never to once a week)	Sponsorship
15	Hing et al.	2014	Self-reported perceived influence of gambling promotions on sports betting (e.g. "How strongly do you agree that X promotions make you want to bet on sports?"); Future sports betting intention for eight sports (5-point Likert; strongly disagree to strongly agree); Future other gambling intention (same measures).	Perceived influence of advertising, actual and intentions	Self-reported frequency of watching eight types of televised professional sport (sports where advertising is most prominent) in the most recent season (7-point Likert; never to daily)	Sponsorship, Aggregate
16	Wardle et al.	2022	Self-reported impact of gambling marketing activities prompting you to spend money on gambling when they otherwise had not planned to (any vs never in the models); Problem Gambling Severity Index groups.	Perceived influence of advertising on behaviour	Cumulative self-reported past month receipt of direct marketing from gambling companies (emails, texts, social media, gambling app notifications, flyers/leaflets); Self-reported following gambling company on social media (yes/no).	Direct, Digital
17	Browne et al.	2019	Self-reported approximate amount bet on race and sports bets in previous 24 hours, and	Gambling over recall period (24	How often they had seen/heard different advertisements and	Direct, TV, Inducements, Aggregate

			intentions to bet in following 48 hours in EMA surveys	hours), actual and intended	inducements in the previous 24-48 hours (never, a few times, often) in EMA surveys	
18	Rockloff et al.	2019	Participants were given money to place bets in an experimental setting and could earn money from placing bets - outcome was riskiness of bets placed (short, medium and long odds).	Bet choice (risk)	Exposure to inducements in experimental conditions; Exposure to simulated sports highlights reels for AFL, cricket and soccer.	Inducements
19	Sproston et al.	2015	Intention to gamble (adolescents) and past year gambling behaviour (adults) on sports, racing, EGMs, other forms of gambling (Likert scale); Problem Gambling Severity Index group.	Gambling over recall period (1 year), intentions, Gambling risk level	Self-reported exposure to marketing for sports and race betting through traditional and digital means; Grouped into none, moderate and high exposure.	Traditional, Digital
20	Schottler Consulting	2012	Self-reported influence of TAB advertising (sports and racing) on unplanned betting (5-point Likert; not at all to very frequently); and also on spending more than planned in the previous 12 months	Perceived influence of advertising on behaviour	Self-reported frequency of viewing TAB advertising (sports and racing) in the past 12 months (5-point Likert; not at all to very frequently).	Aggregate
21	Russell & Hing	2020	Self-reported impact of advertising on increasing or decreasing gambling expenditure.	Perceived influence of advertising on behaviour	Self-reported frequency of seeing gambling advertisements or promotions via 7 channels, and for 13 forms of gambling (including sport and race betting), and five types of promotions (e.g. sign up bonuses, bonus bets).	Aggregate

22	Jenkinson et al.	2023	Self-reported betting on sports and racing in the previous 12 months (less than weekly/more than weekly);	Gambling over recall period (12 months)	Self-reporting whether they had seen advertising, and how often they had seen it in the previous 12 months (4-point Likert; less than weekly to 4 or more times per week); Whether advertising had increased betting, led to impulse betting, changed what someone had bet on (new form), or initiated betting for the first time.	Traditional, Embedded, Digital, Aggregate

**Note:* *PGSI is sometimes used as the key outcome measure, or to separate the effects by gambling risk group; in study by Wardle et al., (2022) we have assumed that the advertising exposure is most likely to be sports-related given that it is a sports betting sample.*

APPENDIX D: DETAILED DATA EXTRACTION TABLE

No.	Authors	Year	Study type (MMAT)	Statistical method	Outcome Variable	Explanatory variable	Coefficient Descriptive	Coefficient	Results summary
1	Houghton & Moss	2020	Quantitative NonRandomised	2-way factorial ANOVA and independent sample ttests	Likelihood of betting (Visual Analogue Scale)	Bet complexity (high/med/low)	F-statistic	F=34.031 p<0.001	The authors find that participants are significantly less likely to bet and would spend lower amounts on high complexity bets compared to medium complexity, and on medium compared to low. They are also significantly more likely to bet on medium complexity bets shown on affiliate accounts compared to operator accounts. There is no significant interaction for money spent.
						Account type (operator/affiliate)	F-statistic	F=5.154 p=0.025	
						Account type*bet complexity	F-statistic	F = 3.781 p=0.025	
					Amount they would spend (£)	Bet complexity (high/med/low)	F-statistic	F=24.837 p<0.001	
						Account type (operator/affiliate)	F-statistic	F=1.494 p=0.225	
						Account type*bet complexity	F-statistic	F=2.695 p=0.07	
2	Noble et al.	2022	Quantitative Descriptive	Logistic mixed regression models (with controls)	Gambled in the last 30 days	Exposure to sports/celebrities ads (Yes/No)	Odds ratio (unadjusted)	OR=1.67 p<0.001	The authors find that adolescents who are exposed to sports gambling advertising are significantly more likely to have bet in the last 30 days, engaged in 'hard' gambling in the last 30 days, and be categorised as at-risk or a 'problem' gambler. However, these do not remain significant after adjusting for confounders (gender, age, money to spend on self, number of known gamblers, socioeconomic disadvantage, perceived school achievement, attended school yesterday and school ID), Despite this, online gambling remains significant. Given that the paper does not categorise types of advert, there is the chance that adolescents are exposed to sports-related advertising online too.
					Gambled in the last 30 days	Exposure to sports/celebrities ads (Yes/No)	Odds ratio (adjusted for confounders)	OR=1.13 p=0.395	
					Engaged in 'hard gambling' in the last 30 days	Exposure to sports/celebrities ads (Yes/No)	Odds ratio (unadjusted)	OR=1.8 p<0.001	
					Engaged in 'hard gambling' in the last 30 days	Exposure to sports/celebrities ads (Yes/No)	Odds ratio (adjusted for confounders)	OR=1.05 p=0.782	
					At risk or 'problem' gambling	Exposure to sports/celebrities ads (Yes/No)	Odds ratio (unadjusted)	OR=1.93 p<0.001	
					At risk or 'problem' gambling	Exposure to sports/celebrities ads (Yes/No)	Odds ratio (adjusted for confounders)	OR=1.04 p=0.773	
3	Roderique-Davies et al.	2020	Quantitative Randomised	2-way factorial ANOVA and independent sample ttests	Urge to gamble (comparison between sports and non-sports students)	Control video (nonsports)	F-statistic	F=0 p=1	The authors find that in the sports group, there are increased urges to gamble when faced with both the professional video with promotion and the amateur video without promotion. This highlights them as a higher risk group, and indicates that there is an innate association between gambling and football. Non-sports students are also urged to gamble when presented with the promotions. There is a significant difference between both groups, indicating that the sports students are a higher-risk group (authors report that they have a higher PGSI).
						Amateur sports video with no embedded promotion	F-statistic	F=10.71 p=0.002	
						Professional sports video with embedded promotion	F-statistic	F=7.87 p=0.007	
4	Russell et al.	2019	Quantitative Descriptive	Two-step zero inflated regression (with controls)	Any betting on microevents	Exposure to gambling advertising	Odds ratio (bivariate)	OR=0.742 p<0.001	The authors found that those with higher self-reported exposure to gambling advertising were significantly less likely to bet on microevents at all. They overall had a lower percentage of total bets made up of micro-bets, but this did not reach statistical significance.
					Any betting on microevents	Exposure to gambling advertising	Odds ratio (multivariate)	OR=0.795 p<0.001	
					Percentage of sports bets that are micro-bets	Exposure to gambling advertising	Odds ratio (bivariate)	OR=0.995 p>0.05	

5	Hing et al.	2019	Quantitative Descriptive	Descriptive statistics (%)	n/a	Relative frequency of exposure	Percentage	n/a	Results have not been reported given the large amount of descriptive statistics reported in the paper. Overall, a substantial minority of participants reported that an advertisement had ever influenced their behaviour. The greatest proportions were for TV advertisements, direct messaging, and betting websites or apps. For race bettors, all types of advertisements and inducements were more likely to prompt larger and increased frequency of betting amongst those reporting any impact. For sports bettors this was true for frequency of betting. Sports bettors reported placing less risky bets as a result of advertisements and inducements.
					n/a	Relative frequency of influence	Percentage	n/a	
6	Hing et al.	2018	Quantitative Descriptive	One-way ANOVA and multiple linear regression	Percentage of bets researched and planned in advance	Exposure to marketing	Standardised coefficient	B=0.04 p=0.068	Results suggest that there is no, or a negative, relationship between self-reported exposure to advertising and the percentage of 'impulse' bets placed before and during the game. However, some inducements might increase the number of 'impulse' bets placed during the game.
					Percentage of bets researched and planned in advance	Average frequency of inducements used	Standardised coefficient	B=-0.19 p=0.852	
					Percentage of bets placed on impulse before the match	Exposure to marketing	Standardised coefficient	B=-0.02 p=0.304	
					Percentage of bets placed on impulse before the match	Average frequency of inducements used	Standardised coefficient	B=-0.19 p<0.001	
					Percentage of bets placed on impulse during the match	Exposure to marketing	Standardised coefficient	B=-0.06 p=0.008	
					Percentage of bets placed on impulse during the match	Average frequency of inducements used	Standardised coefficient	B=0.2 p<0.001	
7	Russell et al.	2018	Quantitative Descriptive	Zero-inflated regression models (with controls)	Intended Expenditure on betting	Exposure to texts (sports bettors)	Regression Coefficient	B=-0.024 p>0.1	For race bettors, only text messaging significantly increased actual expenditure on betting. For sports bettors both emails and texts increased actual expenditure. For intended betting this was for emails only in both groups.
					Intended Expenditure on betting	Exposure to emails (sports bettors)	Regression Coefficient	B=0.465 p<0.001	
					Intended Expenditure on betting	Exposure to texts (race bettors)	Regression Coefficient	B=0.166 p>0.1	
					Intended Expenditure on betting	Exposure to emails (race bettors)	Regression Coefficient	B=0.225 p<0.05	
					Actual expenditure on betting	Exposure to texts (sports bettors)	Regression Coefficient	B=0.832 p<0.001	
					Actual expenditure on betting	Exposure to emails (sports bettors)	Regression Coefficient	B=0.413 p<0.05	
					Actual expenditure on betting	Exposure to texts (race bettors)	Regression Coefficient	B=0.276 p<0.1	
					Actual expenditure on betting	Exposure to emails (race bettors)	Regression Coefficient	B=0.016 p>0.1	
8	Hing et al.	2017	Quantitative Descriptive	Negative Binomial regression (with controls)	PGSI score	Exposure to gambling promotions	Regression Coefficient	B=0.009 p=0.082	The final regression model indicates a non-significant impact of exposure to gambling promotions on PGSI score. However, reporting an increased subjective influence of promotions on sports betting behaviour is positively and significantly associated with PGSI score after controlling for age, gender, sponsorship response, attitudes and approval of (and exposure to) gambling promotions.
					PGSI score	Subjective influence of gambling promotions	Regression Coefficient	B=0.760 p<0.001	
9	Hing et al.	2016	Quantitative Descriptive	Spearman's correlation and Kruskal-Wallis tests	Total PGSI score	Watching live sports at a sporting venue	Spearman's Rho	SpR = 0.26 p<0.001	Results indicate that higher self-reported watching of live sports, and subsequent exposure to advertising, is associated with significant increases in PGSI score.
						Watching televised live sports	Spearman's Rho	SpR=0.22 p<0.001	

10	Di Censo et al.	2023	Quantitative NonRandomised	2 (risk-level) by 4 (inducement type) mixed ANOVAs and hierarchical regression models	PGSI score	Stake back	Standardised coefficient	B=0.22 p=0.006	High-risk gambling scores were significant predictors of PGSI score in the models after controlling for regular gambling, impulsivity, and being male. The sign-up inducement explained the greatest variance in PGSI scores compared to other inducement methods. Findings indicate that those who are at a higher-risk of harms are more likely to believe that inducements exacerbate their gambling problems.
						Sign-up	Standardised coefficient	B=0.3 p<0.001	
						Increased odds	Standardised coefficient	B=0.23 p=0.004	
						Bonus bet	Standardised coefficient	B=0.2 p=0.016	
11	Hing et al.	2015a	Quantitative Descriptive	Summary statistics and hierarchical regression (with controls)	Intention to bet in the next 6 months	Exposure to gambling promotions during televised sport	Standardised coefficient	B=0.107 p<0.01	This study reports that exposure to advertising during televised sports significantly increases the intention of betting in the next 6 months when controlling for a number of potential confounding variables. Descriptive results also indicate that individuals with a higher PGSI score report that advertising increases their frequency, expenditure, and time spent on sports betting to a greater extent than those with a lower PGSI score.
12	Hing et al.	2015b	Quantitative Descriptive	Summary statistics (mean values) and ANOVA	Perceived influence on frequency of sports betting ('problem' gambler)	Exposure to gambling promotions during televised sport	Mean value Likert scale (1=strongly disagree, 5=strongly agree)	Mean=3.5 p<0.001	
					Perceived influence on expenditure on sports betting ('problem' gambler)	Exposure to gambling promotions during televised sport	Mean value Likert scale (1=strongly disagree, 5=strongly agree)	Mean=3.5 p<0.001	
					Perceived influence on time spent betting ('problem' gambler)	Exposure to gambling promotions during televised sport	Mean value Likert scale (1=strongly disagree, 5=strongly agree)	Mean=3.5 p<0.001	
					Perceived influence on frequency of sports betting ('non-problem' gambler)	Exposure to gambling promotions during televised sport	Mean value Likert scale (1=strongly disagree, 5=strongly agree)	Mean=2.2 p<0.001	
					Perceived influence on expenditure on sports betting ('non-problem' gambler)	Exposure to gambling promotions during televised sport	Mean value Likert scale (1=strongly disagree, 5=strongly agree)	Mean=2.1 p<0.001	
					Perceived influence on time spent betting ('nonproblem' gambler)	Exposure to gambling promotions during televised sport	Mean value Likert scale (1=strongly disagree, 5=strongly agree)	Mean=2.1 p<0.001	The descriptive results indicate that 'problem' gamblers report that exposure to advertising has an impact on their frequency, expenditure, and time spent betting on sports. Contrastingly, 'non-problem' gamblers report on average that advertisement do not impact their sports betting. The difference between PGSI groups is significantly different, so the 'problem' gambling group reports a higher impact.

13	LopezGonzalez & Griffiths	2021	Quantitative Descriptive	Kruskal-Wallis and Chi-squared tests	Perceived impact on frequency of sports betting (difference between PGSI groups)	Exposure to gambling promotions	Kruskal-Wallis test statistic	X=247.13 p<0.05	Results indicate that the rank difference in perceived influence of advertisements are statistically significant, so higher risk gamblers report a significantly higher impact of gambling advertising on gambling behaviour compared to lower risk categories. The effect size is noted as large.
14	Johnston & Bourgeois	2015	Quantitative Descriptive	Hierarchical regression (with controls)	Intention to bet with that sponsor	Exposure to gambling sponsorship advertising	Regression coefficient	B=0.11 P<0.05	The results of this study indicate that self-reported exposure to sponsorship advertising in sport is positively associated with intentions to bet with that sponsor after controlling for a number of potential confounding factors. Additionally, perceiving that sponsorship advertising has a 'powerful' effect on oneself is associated with increased intentions to bet in the same model.
					Intention to bet with that sponsor	Perceived 'powerful' impact of gambling sponsorship advertising on oneself	Regression coefficient	B=0.18 p<0.001	
15	Hing et al.	2014	Quantitative Descriptive	Hierarchical regression (with controls)	Intention to bet on sport when 18 years old	Exposure to gambling promotions during televised sport	Correlation (bivariate)	r=0.2 p<0.05	This study indicates that exposure to gambling advertising during sport is significantly correlated with intentions to bet when 18, however this result does not remain significant when controlling for additional factors in a regression model. However, a better attitude towards the sponsor results in an increased intention to bet on sports when 18 years old.
					Intention to bet on sport when 18 years old	Exposure to gambling promotions during televised sport	Regression coefficient (multivariate)	B=-0.112 p>0.05	
16	Wardle et al.	2022	Quantitative Descriptive	Logistic regression (with controls)	Reporting that marketing had prompted unplanned gambling spend (y/n)	Low risk vs 'nonproblem' gambler	Odds ratio	OR=3.31 p<0.001	These results reveal that 'problem' sports gamblers are 17 times more likely than 'nonproblem' gamblers to report that exposure to gambling marketing has prompted unplanned gambling spend. The effects for moderate and low risk are around 3 times more than 'non-problem' gamblers. Additionally, exposure to one type of direct marketing makes participants 3.2 times more likely to report that advertising prompts unplanned gambling spend, and this rises to 5.5 times for more than one exposure to direct marketing. Exposure to a gambling brand on social media increases the likelihood of reporting that advertising has prompted unplanned gambling spend by 2.45 times compared to non-exposure.
						Moderate risk vs 'nonproblem' gambler	Odds ratio	OR=3.41 p<0.001	
						Problem' risk vs 'nonproblem' gambler	Odds ratio	OR=17.01 p<0.001	
						Received one form of direct marketing vs none	Odds ratio	OR=3.2 p<0.001	
						Received more than one form of direct marketing vs none	Odds ratio	OR=5.54 p<0.001	
						Follow a gambling brand on at least one social media platform vs none	Odds ratio	OR=2.45 p<0.05	
17	Browne et al.	2019	Quantitative Descriptive	Linear mixed effects regression models	Intended spend (race)	Aggregate exposure to messaging (advertisements)	Beta coefficient (logistic)	B=0.120 p>0.1	Results show that aggregate exposure to advertisements significantly increases actual spend for sports and race bettors, and excess spend for race bettors. Exposure to advertisements is not significantly associated with intended spend. Other effects show that aggregate exposure to inducements increase actual spend for both sports and race bettors. Specific inducements which have an effect are direct messages, stake-backs and TV advertisements.
					Intended spend (sports)	Aggregate exposure to messaging (advertisements)	Beta coefficient (logistic)	B=-0.151 p>0.1	
					Actual spend (race)	Aggregate exposure to messaging (advertisements)	Beta coefficient (logistic)	B=0.614 p<0.01	
					Actual spend (sports)	Aggregate exposure to messaging (advertisements)	Beta coefficient (logistic)	B=0.553 p<0.01	
					Excess spent (race)	Aggregate exposure to messaging (advertisements)	Beta coefficient (logistic)	B=0.374 p<0.01	
					Excess spend (sports)	Aggregate exposure to messaging (advertisements)	Beta coefficient (logistic)	B=0.227 p>0.1	

18	Rockloff et al.	2019	Quantitative NonRandomised	Wilcoxon Signed Rank Test, ANOVA, Chi-sqd test	Odds selected (short, medium, long)	Inducements vs no inducement	Mean value	M=+ p<0.05	The results show that participants tended to choose longer, more risky, odds when an inducement was present compared to when there was no inducement present. The only inducement that showed an independent effect was Cash Rebate.
						Cash Rebate vs no inducement	Mean value	Mcb=1.66, Mni=1.55 p<0.05	
19	Sproston et al.	2015	Quantitative Descriptive	Logistic regression (with controls)	Gambled regularly on sports (adults)	Moderate exposure to sports digital marketing (compared to no exposure)	Odds ratio	OR=1.47 p<0.01	These results suggest that exposure to digital sports betting marketing is associated with regular sports betting in adults. Exposure to race betting marketing on both digital and traditional channels is also associated with gambling regularly on racing, EGMs, and other activities. In the sample of adolescents, only exposure to race marketing via digital means was significantly associated with likelihood of gambling on another activity.
					Gambled regularly on sports (adults)	High exposure to sports digital marketing (compared to no exposure)	Odds ratio	OR=3.06 p<0.01	
					Gambled regularly on horse or greyhound racing (adults)	Moderate exposure to traditional racing marketing (compared to no exposure)	Odds ratio	OR=3.07 p<0.01	
					Gambled regularly on horse or greyhound racing (adults)	High exposure to traditional racing marketing (compared to no exposure)	Odds ratio	OR=4.11 p<0.01	
					Gambled regularly on EGMs (adults)	Moderate exposure to digital racing marketing (compared to no exposure)	Odds ratio	OR=0.86 p<0.01	
					Gambled regularly on EGMs (adults)	High exposure to digital racing marketing (compared to no exposure)	Odds ratio	OR=1.62 p<0.01	
					Gambled regularly on EGMs (adults)	Moderate exposure to traditional racing marketing (compared to no exposure)	Odds ratio	OR=2.00 p<0.05	
					Gambled regularly on EGMs (adults)	High exposure to traditional racing marketing (compared to no exposure)	Odds ratio	OR=1.72 p<0.05	
					Gambled regularly on another activity (adults)	Moderate exposure to digital racing marketing (compared to no exposure)	Odds ratio	OR=1.22 p<0.05	
					Gambled regularly on another activity (adults)	High exposure to digital racing marketing (compared to no exposure)	Odds ratio	OR=2.07 p<0.05	
					Gambled regularly on another activity (adults)	Moderate exposure to traditional racing marketing (compared to no exposure)	Odds ratio	OR=1.69 p<0.05	
					Gambled regularly on another activity (adults)	High exposure to traditional racing marketing (compared to no exposure)	Odds ratio	OR=1.69 p<0.05	
					Likely to gamble on another activity (adolescents)	Moderate exposure to digital racing marketing (compared to no exposure)	Odds ratio	OR=5.00 p<0.05	

					Likely to gamble on another activity (adolescents)	High exposure to digital racing marketing (compared to no exposure)	Odds ratio	OR=14.28 p<0.05	
20	Schottler Consulting	2012	Quantitative Descriptive	Summary statistics (mean values)	Unplanned betting	Self-reported impact of TAB (sports) advertising	Mean value Likert scale (1= not at all , 5=very frequently)	M=1.1 range=1.1-1.9	These results indicate that participants were not likely to rate that exposure to sports betting advertising impacted unplanned spend on gambling. Results split by 'problem' gambling group also revealed low mean Likert scores (<2). However, risk of 'problem' gambling was a significant predictor of self-reported advertising influence, but the correlation was low.
					Unplanned gambling spend	Self-reported impact of TAB (sports) advertising	Mean value Likert scale (1= not at all , 5=very frequently)	M=1.1 range=1.1-1.6	
				Stepwise regression	Self-reported influence of advertising	Risk of 'problem' gambling	Partial correlations	r=0.37 p<0.001	
21	Russell & Hing	2020	Quantitative Descriptive	Summary statistics (% Likert, McNewmar-Bowker test)	Self-reported influence of advertising on gambling expenditure	Before lockdown	% reporting 'decreased a little due to advertising'	3.8%	In general, participants reported that gambling advertising did not impact their expenditure ('neither increased nor decreased') before or during lockdown. However, participants were significantly more likely to report that advertising during lockdown led to a decrease in their expenditure on gambling. This was a period when advertising temporarily reduced for usual sports betting, given the pause in live sports that occurred during the initial lockdown.
					Self-reported influence of advertising on gambling expenditure	During Lockdown	% reporting 'decreased a little due to advertising'	6.6%	
					Self-reported influence of advertising on gambling expenditure	Comparison before and during lockdown	McNewmar-Bowker test	MB=97.53 p<0.001	
22	Jenkinson et al.	2023	Quantitative Descriptive	Summary statistics (% Likert)	Self-reported increased betting	Exposure to any advertising	Percentage	34%	These descriptive results show that 20-30% of respondents report that exposure to any type of advertisement for sports betting influences their betting behaviour, including initiating betting for the first time and betting on impulse. More detailed results in the report show that younger people and those at more risk of harm were more likely to report these effects (e.g. 10% in the lower lower-risk vs 50% in the higher-risk groups).
					Self-reported bet on impulse	Exposure to any advertising	Percentage	29%	
					Self-reported change betting/try something new	Exposure to any advertising	Percentage	28%	
					Self-reported initiate betting for the first time	Exposure to any advertising	Percentage	21%	

APPENDIX E: DETAILED QUALITY ASSESSMENT TABLE

Study Type (MMAT)	*Paper No.*	**Main Q1**		**Main Q2**		Q1		Q2		Q3		Q4		Q5	
**Quantitative Randomised (experimental)**		**Are there clear research questions?**	*Comments*	**Do the collected data allow the authors to address the research questions?**	*Comments*	**Is randomisation appropriately performed?**	*Comments*	**Are the groups comparable at baseline?**	*Comments*	**Are there complete outcome data?**	*Comments*	**Are outcome assessors blind to the intervention being provided?**	*Comments*	**Did the participants adhere to the assigned intervention?**	*Comments*
	3	Y	Clear aim and hypotheses reported at the end of the introduction section.	?	The small sample size and the fact that the researchers could not find a professional football control video mean that the data may not be as able to answer the research question. However, the methods are novel and perhaps a larger (nonpilot) study would be useful for confirming results.	Y	The authors use block randomisation which aims to randomise participants into groups of equal sizes.	N	The groups differ in PGSI score, although the authors explain that this is purposeful since they wish to understand the effect on those with higher vs lower risk of gambling harms.	?	The authors do not report any missing data, but do not clarify response rates.	?	The authors do not mention this but I assume that they were not blind to it given that they performed the randomisation and invited students into the experiment.	Y	All 60 participants took part in the experiment.
**Quantitative Nonrandomised (experimental)**		**Are there clear research questions?**	*Comments*	**Do the collected data allow the authors to address the research questions?**	*Comments*	**Are the participants representative of the target population?**	*Comments*	**Are the measurements appropriate regarding both the outcome and intervention (or exposure)?**	*Comments*	**Are there complete outcome data?**	*Comments*	**Are the confounders accounted for in the design and analysis?**	*Comments*	**During the study period, is the intervention administered (or exposure occurred) as intended?**	*Comments*
	1	Y	Clear hypotheses stated on page 390.	Y	The author's measure individual response to social media advertising and compare results between adverts on operator and affiliate accounts, which relates directly to their hypotheses.	N	Whilst it is understandable why the authors used these sampling methods, the final sample has much higher rates of moderate-risk and highest-risk gambling, as well as a much higher percentage of individuals with university education.	?	The PGSI is a validated clinical measure of gambling behaviour, and is one of the most widely used in the gambling literature. Whilst VAS are commonly used to rate pain, there is no indication whether this is appropriate for the current study. The mock advertisements are not likely to reflect real-life advertisements due to budget restrictions, but the authors acknowledge this as a limitation.	?	The authors impute data but do not give a reason why this is appropriate, and do not address/test whether it may cause bias.	?	There is some mention of demographic and gambling characteristics, but the authors do not explain how/whether the method they use controls for these.	Y	The authors have 100/145 participants with at least 50% response rate for the advertisements.

	10	Y	The authors clearly state their aims and objectives under 'The Present Study' section	Y	The authors have control over exposure to advertising inducements, and measure individual perceived impact immediately after exposure.	N	The authors use a prior sample size calculation and a panel to recruit participants, but their sampling method is non-representative because authors are interested in individuals who have prior experience with sports betting.	Y	The authors provide a detailed explanation of the development and appropriateness of their measurements, quoting Cronbach's alpha statistics.	?	There were a large number of individuals who did not complete the survey, and 49 were excluded at a later date due to issues with their responses. However, using an online panel tends to reduce nonresponse since these individuals are signed up to complete surveys.	Y	The use of hierarchical regression models with some controls has strength. The authors may have included additional demographic and gambling-related controls, if they had the data available.	?	A large number of individuals did not complete the survey and this may bias results if this is for reasons correlated with their betting behaviour.
	18	Y	The authors state clear research questions at the end of the introduction section.	Y	The authors utilise experimental methods to measure the effect of exposure to inducements on the selection of odds by surveying participants, whilst controlling for exposure directly.	N	This study oversamples higher frequency gamblers and as a result there is an over-representation of 'problem' gamblers in the sample. However, the authors acknowledge this. This is also common in the gambling advertising literature to ensure that sufficient numbers of individuals are in each gambling risk group (the authors specifically tested for differences by PGSI group).	Y	The authors use previously explored inducement types, a valid measure of gambling behaviour (PGSI) and are able to directly control for exposure to advertising given the experimental setup.	?	The authors do not discuss any missing data, only that participants were dropped because they did not meet inclusion criteria (e.g. place of residence), or did not give complete answers at the soft launch.	Y	The use of an experimental setup helps control for potential confounders and makes the study more internally valid.	Y	The experimental setup means that the authors had full control over exposure to the different types of inducement.
**Quantitative Descriptive (observational)**		**Are there clear research questions?**	*Comments*	**Do the collected data allow the authors to address the research questions?**	*Comments*	**Is the sampling strategy relevant to address the research question?**	*Comments*	**Is the sample representative of the target population?**	*Comments*	**Are the measurements appropriate?**	*Comments*	**Is the risk of nonresponse bias low?**	*Comments*	**Is the statistical analysis appropriate to answer the research question?**	*Comments*

	2	Y	The authors clearly state their aims at the end of the introduction section.	Y	The authors collect data on (1) adolescents selfreported exposure to different types of advertisements (2) their gambling behaviour, including gambling in the past month and at risk/'problem' gambling.	Y	Yes, they are able to recruit a random sample that is more representative of the population of interest.	Y	The authors use a large, randomised, and weighted study on adolescents' alcohol and drug use, and gambling.	?	The gambling questions were developed through an interactive process including: literature search, expert advice, and pilot testing. Students were given a definition of gambling before answering the questions. The authors used a reliable and valid measure of gambling behaviour (DSM-IV), and justified their choice of using a dichotomous response option. Authors used and adapted an already tested measure of advertising exposure (Hing et al 2014). However, the categorisation of advertising exposure did not acknowledge any cross-over between advertising types (e.g. sports advertisements can also be online and TV).	Y	The authors are already using a large, wellestablished survey of adolescent behaviours.	Y	The authors use logistic models which are appropriate and easy to interpret. They also adjust for a number of important demographic and gambling-related confounders. The sample size is also an advantage of this study.
	4	Y	The authors clearly state their aims at the end of the introduction section.	Y	Whilst they cannot comment on causality, the data does allow the authors to investigate their research hypotheses.	Y	The authors explain that their sampling strategy is purposeful and allowed them to recruit sufficient numbers of respondents at varying levels of 'problem' gambling severity.	N	The authors state that this is a convenience sample and that the sample is not representative of the general population of Australia, and they explain why this was done.	?	The authors use well validated measures and reference Cronbach's alpha statistics in their methodology section. However, their measure of gambling advertising was selfreported and only ranged from 'never' to 'almost always'. They were also measuring a type of betting that is illegal in Australia.	Y	The authors do not discuss response rates. However, they have used an online panel which usually increases response rates, and the quality of data collected.	?	Whilst the authors use appropriate statistical models, the main aim of the study wasn't to measure the relationship between advertising and behaviour. The association measured is potentially biased.
	5	Y	The authors explain their overall aim at the end of the introduction section.	?	Whilst the data does answer the question, it is very descriptive data.	Y	Over-sampling higher risk gamblers is common in the gambling literature to ensure that there are enough participants in each risk category.	N	The authors acknowledge this as a limitation, but explain why this is the case.	Y	Whilst exposure to advertising is selfreported, the authors' use EMA methods which can minimise recall bias by measuring exposure as close to the exposure time as possible.	?	The authors' do not report the percentage of surveys completed by all participants.	N	There is no statistical analysis, this is a descriptive study.

	6	Y	The authors clearly state their aims and hypothesis at the end of the introduction section.	Y	The authors collect selfreported data on advertising exposure, watching of sports, uptake of inducements and percentage of bets placed on 'impulse' before and during the game.	Y	Over-sampling higher risk gamblers is common in the gambling literature to ensure that there are enough participants in each risk category.	N	The authors explain why they have collected a nonrepresentative sample: cost considerations and to ensure that there are sufficient numbers of respondents in each gambling risk group.	?	The measurement of betting relies on the respondent understanding which bets they have placed on ' impulse' in the form of a percentage of total bets. This is quite a subjective question and is most likely subject to recall bias. The authors also control for both watching of sport, and exposure to advertisements which may be correlated. However, they use other validated measures such as 'problem' gambling severity, and report Cronbach's alpha for a number of their measures.	Y	The authors do not discuss response rates. However, they have used an online panel which usually increases response rates, and the quality of data collected.	?	The regression models control for a number of important confounding demographic and gambling variables, but the measures used might not be accurately measuring what they intend to.
	7	Y	The authors clearly state their aims and hypothesis at the end of the introduction section.	Y	The authors use novel EMA methods to collect real-time data on exposure to direct advertising, and expenditure on betting. They also collect actual direct messages from individuals to examine their content.	Y	Over-sampling higher risk gamblers is common in the gambling literature to ensure that there are enough participants in each risk category.	N	The authors collected a small convenience sample due to budget constraints.	Y	The use of EMA helps to reduce recall bias in the measurements, especially in exposure to advertising by collecting the data as close to the exposure as possible. The authors use a widely used measure of gambling behaviour (PGSI).	?	The sample is very small and the authors do not report how many individuals complete each survey, but do report that 65% completed 6/7 surveys. The percentage of direct messages forwarded to the authors' is variable and can be low for sports bettors.	Y	They use zeroinflated regression models with control variables to estimate effects for two groups of bettors (race and sports). Their use of EMA also increases the ecological validity of the models, and reduces recall bias. They also controlled for individual random effects to account for differences in individual betting, and for PGSI score which strengthen the models.
	8	Y	There are clear aims and hypotheses at the end of the introduction section.	Y	The authors collect data on 'problem' gambling scores, and exposure to gambling promotions (using a proxy measure) to estimate this relationship. They also collect data on confounding variables such as age and gender.	Y	Over-sampling higher risk gamblers is common in the gambling literature to ensure that there are enough participants in each risk category.	N	The authors explain why the sample is notrepresentative (this was not their aim).	Y	The authors clearly explain each measure used and provide Cronbach's alpha statistics for each. The use of a proxy measure for advertising exposure is useful for overcoming issues with recall of advertising exposure, but it is also still a self-reported variable so may suffer bias.	Y	The authors have used a panel to recruit participants which enhances completeness of the data.	Y	The use of Negative Binomial regression with control variables is a strength of this study. Other variables might have been useful to control for, such as other gambling behaviours, if they were available in the dataset.

	9	Y	The authors clearly state their aims at the end of the introduction section.	?	The results are very descriptive, but they do answer the question.	Y	The authors deliberately oversample 'at least fortnightly' bettors to ensure sufficient numbers of 'problem' and at-risk gamblers.	N	See previous comment. The authors note that only 13% of the Australian population gambles on sport, so gaining a random sample of sports bettors is not feasible (needs purposive methods).	?	The use of a proxy measure for advertising exposure is useful for overcoming issues with recall of advertising exposure, but it is also still a self-reported variable so may suffer bias. It also may not be directly measuring exposure to advertising (although they are likely strongly correlated). The outcome variable is a well validated measure of gambling behaviour.	Y	The authors do not discuss response rates. However, they have used an online panel which usually increases response rates, and the quality of data collected.	?	Looking at descriptive statistics is useful, but it doesn't control for potential confounding variables in the relationship between advertising and behaviour. So it is hard to establish causality for each single risk factor.
	11	Y	The authors state clear hypotheses at the end of the introduction section.	Y	The authors collect data on self-reported perceived impact of advertising, and watching live sports (proxy for advertising exposure). They use descriptive models and hierarchical regression models with controls to explore this relationship.	Y	The authors use an online panel to collect a large sample of data.	Y	Online panels are representative of the population by gender and metro/non-metro location. People aged 45-74 were only slightly overrepresented.	Y	The authors provide a clear table with explanations of where the measures are derived from, with Cronbach's alpha coefficients where applicable.	Y	The authors do not discuss response rates. However, they have used an online panel which usually increases response rates, and the quality of data collected.	Y	However they could have controlled for additional demographic characteristics given that this was a large, detailed sample with information on income/area of residence/age etc…
	12	Y	There is a general aim stated at the end of the introduction section.	?	The results are very descriptive, but they do address the overall aim.	Y	Purposive samples are common in the gambling advertising literature, and the authors acknowledge why they have chosen to do this (ensure sufficient numbers in each risk group). They also explain why and how they collected an additional sample from a pool of individuals who had previously completed a study.	N	But the authors explain why they have chosen this sample. They want to look at differences between PGSI groups, so need to ensure that there are sufficient numbers in each group (oversampling higher risk gamblers).	?	The authors have used a widely used and validated measure of gambling behaviour (PGSI). However they do not reference the validity of the other measures used. These appear to be the same ones referenced in the above paper (Hing et al., 2015b).	Y	The use of a research panel generally increases response rates and the quality of data.	?	Whilst the authors acknowledge that the study provides modest and preliminary knowledge about the topic, this is a limitation of the paper. The results are descriptive, rely on self-report and do not control for other confounding factors.
	13	Y	The paper clearly states a number of aims in the section titled 'The Present Study'	Y	The authors collect data on perceived impact of advertising on behaviour, and the PGSI score of each individual which allows them to test the relationship between the two.	Y	The authors use a panel which helps to reduce missing data/non-response, and over-sample higher risk gamblers.	N	The sample has a higher percentage of males, and appears to have a high percentage of those with a bachelor's degree or higher (although this common in online panel samples). The authors explain why they have sampled this way (over-sample higher risk gamblers).	Y	Each measure is welldescribed and has an associated Cronbach's alpha statistic.	Y	The use of a research panel generally increases response rates and the quality of data.	?	The analysis is directly related to the aims of the study and uses non-parametric statistical tests to explore whether Reponses between PGSI groups are statistically significant. Given that the authors have demographic information, they could have used regression models to control for all factors in the model at the same time to improve the

															robustness of their results.
	14	Y	The authors present clear hypotheses (relevant ones under 'intentions to gamble with sponsors')	Y	The authors collect data on sponsorship exposure, intentions to use that sponsor, and a number of other demographic and gambling characteristics to explore this relationship.	Y	Their use of a quota sample allows the authors' to look at the impact of this relationship in a broader context (not just on high frequency sports bettors).	?	The authors use a panel and quota sampling methods which should improve sample representativeness, but they do not discuss how the sample compares to the population.	?	The authors use previously tested measure of gambling intentions and quote Cronbach's alpha statistics for internal reliability. However, their measure of gambling involvement and exposure to advertising do not reference a previously used measure or a measure of internal reliability.	?	Although the authors use an online panel, they report an initial response rate of 24% with a dropout rate of 11%. However, they do collect 511 useable responses.	Y	The authors use hierarchical regression models and control for a number of potential demographic and gambling-related characteristics in the model.
	15	Y	The authors state clear hypotheses at the end of the introduction section.	Y	The authors collect selfreported survey data on adolescent exposure to advertising (proxied by sports watching) and on their intentions to bet on sports at 18 years old.	?	The authors use a panel to collect a general sample of adolescents to try and make the sample more representative. However, the authors cannot guarantee the the final sample is representative of the population of interest, but it also does not over-sample those more interested in sports watching.	N	The sample is representative in terms of gender, the authors' state that it might not be representative based on other variables. The authors' cannot comment on the representativeness compared to the general population.	Y	Each measure is welldescribed and has an associated Cronbach's alpha statistic.	Y	The use of a research panel generally increases response rates and the quality of data.	Y	The authors use hierarchical regression models with controls.
	16	Y	The authors' clearly state their aims in their abstract and introduction.	Y	The authors have a large dataset measuring unplanned gambling spend prompted by marketing, exposure to, and awareness of, gambling marketing, and PGSI score.	Y	The authors use a large dataset of British sports bettors from an online survey. Participants are recruited via YouGov which is a trustworthy and representative survey platform.	Y	The authors weight the sample by age, sex, and region with respect to the population profile of Great Britain.	Y	Each measure is welldescribed and comes from the larger cohort survey, which has likely undergone rigorous testing prior to collection of data. Cronbach's alpha is reported for PGSI score.	Y	The authors are using a sample from a cohort study recruited via an online platform which likely increases response rates and completeness of data.	Y	The authors use logistic regression models with a number of important control variables (sex, age, educational attainment, employment and deprivation).

	17	Y	There is a clear aim stated at the end of the introduction section.	Y	The authors use novel EMA methods to collect real-time data on exposure to advertisements and inducements and intended and actual gambling spend over a period of time.	Y	The authors targeted a sample of sport and race bettors in Australia since they were a group of interest.	N	The sample has an over-representation of higher risk gamblers which is often observed in internet panel samples, and also amongst purposive samples of sports bettors. There was attrition in the EMA surveys which means the final sample may differ slightly to the general population (differential attrition by age for race bettors).	Y	Using EMA ensures that data is collected as close as possible to time of exposure or time of expenditure on gambling, so the risk of recall bias is reduced. The surveys used a list of advertisements from a previous national study.	N	There was significant attrition in this EMA surveys, which potentially differed by age in the race betting sample. These types of surveys are a higher burden on participants, which may explain this.	Y	The authors carefully explain their choice of methods, and use linear mixed models with controls (e.g. Saturdays) to measure the effects.
	19	Y	The authors state a clear objective at the beginning of chapter 5 (online survey).	Y	The authors collect a large sample of data on self-reported exposure to sports and race betting advertising and gambling behaviour.	Y	The authors use purposive sampling methods to recruit using an online panel.	N	The authors explain that it was not within their budget to get a randomised representative sample since this would require too large a sample size to ensure there were sufficient numbers in each group.	?	Some measures are based on measures used in a previous study. There are no references to statistics to test the validity or reliability of the measures.	Y	The authors recruit using an online panel which should maximise completeness of data.	Y	The authors use logistic regression models with controls for other advertising-related and demographic variables.
	20	Y	The authors state the broad aim of the quantitative research segment in their introduction and at the beginning of the relevant chapter.	?	Whilst the authors collect data on selfreported unplanned gambling behaviour, this might be difficult for participants to answer correctly.	Y	The authors use a weighted sample which improves the representativeness of the sample (in terms of it being comparable to the betting population in New Zealand).	N	Whilst the authors do weight the sample, they acknowledge the use of a panel and the non-generalisability of the sample to the entire New Zealand population.	?	There is no clarification of where the measures used are derived from.	Y	The use of a research panel generally increases response rates and the quality of data.	?	For the relationship between advertising and behaviour there are only summary statistics (mean Likert values). The stepwise regression models are only used for looking at predictors of selfreported influence of advertising on behaviour.
	21	Y	The authors report four clear research questions in the background section of the report.	Y	The authors use an online survey to collect a large sample of data on self-reported exposure to advertising and impact of advertising on expenditure before and during the initial lockdown period. The defined (and important) periods may have helped participant recall.	Y	The use of purposive sampling methods using an online panel and participants from a previous study allowed the authors to collect a much larger sample of data.	N	The authors used purposive methods because they were interested in recruiting regular bettors, and therefore the sample was not representative of the population (although the authors have reasons for this).	?	There is no clarification of where the measures used are derived from, but they appear to use a Likert scale when asking respondents to report the impact of advertising on their expenditure.	Y	The use of a research panel and participants who had previously completed a study likely increases response rates and the quality of data.	?	The results are descriptive, although the authors acknowledge this in the report.

	22	Y	Though there are no specific research questions reported (the document is a research snapshot summary), there are clear aims under the 'our survey' section.	Y	Despite the descriptive nature of the results, the collected data do allow the authors to understand exposure and impact of gambling advertising overall and within specific subgroups of interest.	Y	The authors collect a large general community sample which is aligned to population parameters. They are able to look at different subgroups including age, gender and gambling risk group.	Y	The sample is representative in terms of gender, age and location of residence (metro vs non-metro). The sample was aligned with BAS population parameters.	?	There is no clarification of where the measures used are derived from, but they use a Likert scale ('strongly agree' to 'strongly disagree').	Y	The authors collect survey data with a large research company and online research unit (panel) which improves completeness of the data.	?	The results are descriptive, but the authors are able to divide the results by subgroup which is a strength of the study.

**Response options: Y (yes), N (no), ? (can't tell)*
